# Use of the ‘Supporting Treatment and Appropriate Medication in Paediatrics’ (STAMP) Criteria in Young People (YP) With an Intellectual Disability (ID) And/or Autism Spectrum Disorder (ASD) at Child and Young People’s Services (CYPS) St. Luke’s Hospital, Malta

**DOI:** 10.1192/bjo.2026.11664

**Published:** 2026-06-30

**Authors:** Nicole Mifsud, Noemi Zammit Cortis, Jean-Pierre Giorgio

**Affiliations:** 1 Mater Dei Hospital, Msida, Malta; 2 Mental Health Services, Attard, Malta

## Abstract

**Aims:**

This audit assessed whether psychotropic prescribing practices for young people (YP) with Intellectual Disability (ID) and/or Autism Spectrum Disorder (ASD) under the care of the ID Team aligned with STAMP principles. It also examined the proportion of patients prescribed psychotropic medication, the class of psychotropic medication used, and the quality of documentation regarding multidisciplinary input, medication reviews, behavioural interventions, and involvement of YP and their carers in the decision-making process.

**Methods:**

All YP under 18 years with a diagnosis of ID and/or ASD receiving care from the ID team, were included. Electronic Patient Records were reviewed with demographic data, comorbid mental and physical health conditions, psychotropic medications prescribed (antipsychotics, antidepressants, mood stabilisers, anxiolytics, and Attention Deficit Hyperactivity Disorder (ADHD) medication), and documentation against STAMP criteria included. The latter included evidence of a clear clinical indication for medication use, use of non-pharmacological interventions, timely medication reviews, patient and carer involvement, and plans for medication reduction or discontinuation.

**Results:**

The cohort comprised 120 patients with a mean age of 14.7 years; 86 were male and 34 female. Within the ID clinic, ASD was diagnosed in 91 patients (75.83%) and common comorbidities included ADHD (60%), anxiety disorders (14.17%), and epilepsy (10.83%).

113 patients (94.2%) were prescribed psychotropic medications. Antipsychotics were most commonly prescribed (n=70, 58.3%), followed by ADHD medications (n=56, 46.67%), antidepressants (n=54, 45%), anxiolytics (n=47, 39.17%), and mood stabilisers (n=18, 15%). A clear clinical indication was documented in 100% of cases.

Non-pharmacological interventions were widely used (n=115, 95.8%), with school-based support in 88 patients (76.5%), psychology assessments/referrals in 42 (36.5%), behavioural therapy in 35 (30.4%), occupational therapy in 28 (24.3%), and psychotherapy in 24 (20.9%). Multidisciplinary team (MDT) involvement was documented in 117 cases (97.5%), and parent or carer involvement in care planning was recorded in all patients (100%). Medication reviews within the previous 12 months were documented for 117 patients (97.5%), and reduction or tapering plans were present for 81 patients (67.5%).

**Conclusion:**

This audit highlights a high proportion of psychotropic medication use, particularly antipsychotics. While multidisciplinary involvement was prominent, there was little mention of positive behavioural therapy and behavioural plans used to guide judicious prescribing. Further emphasis on the importance of behavioural strategies throughout the department would help improve awareness and responsible prescribing practices.

